# Effect of Heat Treatment on Structure of Carbon Shell-Encapsulated Pt Nanoparticles for Fuel Cells

**DOI:** 10.3390/nano14110924

**Published:** 2024-05-24

**Authors:** Khikmatulla Davletbaev, Sourabh S. Chougule, Jiho Min, Keonwoo Ko, Yunjin Kim, Hyeonwoo Choi, Yoonseong Choi, Abhishek A. Chavan, Beomjun Pak, Ikromjon U. Rakhmonov, Namgee Jung

**Affiliations:** 1 Graduate School of Energy Science and Technology (GEST), Chungnam National University, 99 Daehak-ro, Yuseong-gu, Daejeon 34134, Republic of Korea; haki030899@o.cnu.ac.kr (K.D.); schougule@o.cnu.ac.kr (S.S.C.); mjh9780@o.cnu.ac.kr (J.M.); kkw00000@o.cnu.ac.kr (K.K.); yunjinkim1994@o.cnu.ac.kr (Y.K.); snow7780@o.cnu.ac.kr (H.C.); gubongman@o.cnu.ac.kr (Y.C.); abhishek@o.cnu.ac.kr (A.A.C.); ssansai@o.cnu.ac.kr (B.P.); 2 Department of Power Supply, Tashkent State Technical University, Tashkent 100095, Uzbekistan

**Keywords:** proton exchange membrane fuel cell, Pt catalyst, annealing, H_2_ content, flow rate, carbon shell, oxygen reduction reaction, durability

## Abstract

Polymer electrolyte membrane fuel cells (PEMFCs) have attracted much attention as highly efficient, eco-friendly energy conversion devices. However, carbon-supported Pt (Pt/C) catalysts for PEMFCs still have several problems, such as low long-term stability, to be widely commercialized in fuel cell applications. To address the stability issues of Pt/C such as the dissolution, detachment, and agglomeration of Pt nanoparticles under harsh operating conditions, we design an interesting fabrication process to produce a highly active and durable Pt catalyst by introducing a robust carbon shell on the Pt surface. Furthermore, this approach provides insights into how to regulate the carbon shell layer for fuel cell applications. Through the application of an appropriate amount of H_2_ gas during heat treatment, the carbon shell pores, which are integral to the structure, can be systematically modulated to facilitate oxygen adsorption for the oxygen reduction reaction. Simultaneously, the carbon shell functions as a protective barrier, preventing catalyst degradation. In this regard, we investigate an in-depth analysis of the effects of critical parameters including H_2_ content and the flow rate of H_2_/N_2_ mixed gas during heat treatment to prepare better catalysts.

## 1. Introduction

Polymer electrolyte membrane fuel cells (PEMFCs) have attracted much attention as eco-friendly energy conversion technologies that emit no CO*_2_* and can immediately transform chemical energy into electrical energy [1,2,3]. As a cathode catalyst in PEMFCs, Pt is commonly employed for oxygen reduction reactions (ORRs) since it shows the highest activity among all pure metals [4,5]. However, conventional carbon-supported Pt nanoparticles (Pt/C) still face several challenging issues that hinder their practical use in fuel cell applications. In detail, structural deterioration such as the agglomeration, dissolution, and detachment of Pt nanoparticles that occurs under the harsh operating conditions of a fuel cell considerably decreases the ORR activity of the Pt/C catalyst due to the loss of active sites [6,7,8,9].

Over the past few decades, researchers have investigated several synthetic approaches to improve the long-term stability of Pt-based catalysts. In particular, the extensive documentation of core/shell- or Pt skin-structured nanoparticle advancement is well documented within the academic literature. However, obtaining catalysts that exhibit both high durability and activity remains a challenging task when only modifying the metal nanoparticle structures. Recently, to improve the durability of metal nanoparticles, further studies suggested the interesting idea of encapsulating metal nanoparticles with a chemically stable carbon shell through polymer coating/graphitization or chemical vapor deposition (CVD) processes [10,11,12,13,14,15,16,17,18,19,20,21,22]. In CVD, methane (CH_4_) or carbon monoxide (CO) gas provides the carbon source to coat a carbon layer on the surface of metal nanoparticles [23]. However, the decomposition of the carbonaceous gas and the formation of the carbon shell are too slow to avoid the increased size of the metal nanoparticles during the high-temperature annealing process, leading to the reduced electrochemically active surface area (ECSA) [24,25,26]. In contrast, the bulky structure of polymers makes it difficult to effectively regulate the pore structure and thickness of the carbon layer during the polymer coating/graphitization process [27]. Hence, to circumvent the above-mentioned drawbacks of the processes, more effective approaches that allow for the fine control of the structure of carbon shell-encapsulated metal nanoparticles should be developed.

According to our previous studies [28], the preparation of carbon-incorporated metal nanoparticles followed by an annealing process can effectively form an ultrathin carbon shell on the surface of metal nanoparticles without changing the particle size. In detail, a metal precursor containing organic ligands is used in pyrolytic synthesis to generate a carbon source which serves to encapsulate the nanoparticles during the post-annealing process. Furthermore, the thickness and porosity of the carbon layer can be modulated by simply adjusting the heat treatment parameters such as annealing gas composition and temperature, rationally modulating the number of active sites without changing the particle size.

**Figure 1:**
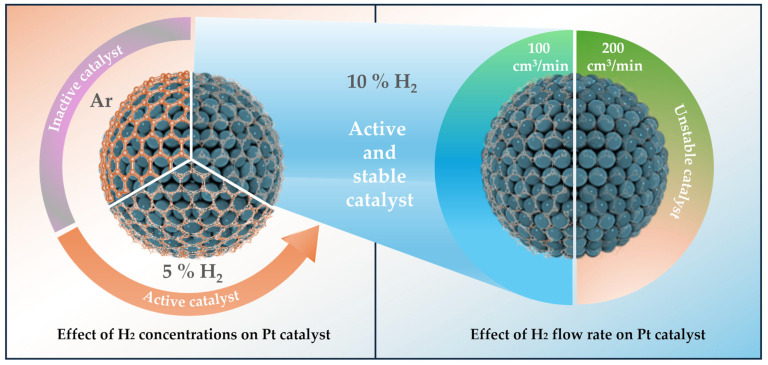
A schematic diagram showing the strategy to control the carbon shell structure by using different gas atmospheres during the annealing process for Pt catalysts.

Although many studies have been conducted on the effect of annealing gas composition and temperature, the effect of gas flow conditions during heat treatment has not yet been fully explored. In this work, we deeply investigate the annealing gas conditions as a key factor in developing finely tuned carbon shell structures. Specifically, we investigate the effect of H_2_ gas concentration and flow rate to fabricate highly active and durable carbon shell-encapsulated Pt nanoparticles on carbon supports (Pt@C/C), as shown in Figure 1. In fact, when heat-treated in a H_2_ gas atmosphere at high temperature, Pt@C/C catalysts can have porous carbon layers on the Pt surface, exposing more active sites for the ORR. We expect that this study will provide insight into the effect of H_2_ gas treatment on the electrochemical properties of carbon shell-encapsulated metal nanoparticles for PEMFCs.

## 2. Materials and Methods

### 2.1. Synthesis of Carbon Shell-Formed Pt Catalysts

In order to create Pt nanoparticles supported on carbon with a carbon shell, 0.1 g of carbon black Vulcan XC-72 (Cabot Inc., Boston, MA, USA) was dispersed in a solution that contained 120 mL of 90% pure 1-octadecene (Sigma-Aldrich Inc., Burlington, MA, USA) and 5 mL of 70% pure oleyamine (Cabot Inc., Boston, MA, USA) in a three-necked round-bottom flask. To guarantee uniform suspension, this combination was subjected to 20 min of sonication. Separately, a solution was made in a vial using ultrasonication for 20 min. It contained 0.0525 g of Pt(acac)_2_ (97% purity, Sigma-Aldrich Inc., Burlington, MA, USA), 40 mL of 1-octadecene, and 5 mL of oleyamine. Upon mixing the precursor with the carbon suspension in the flask, the resulting solution underwent sonication for an additional 5 min. Subsequently, the solution was subjected to heating at 120 °C for a duration of 1 h within an Ar-saturated atmosphere to eliminate trace amounts of impurity water. Following this, the solution temperature was increased to 300 °C and maintained for 2 h to facilitate the pyrolysis of the metal precursor, after which it was reduced to 80 °C. Then, the solution underwent filtration and thorough washing with abundant n-hexane (95% purity, Samchun Pure Chemicals, Daejeon, Republic of Korea) and ethanol (95% purity, Samchun Pure Chemicals, Daejeon, Republic of Korea) solutions. Finally, the resulting as-prepared (ASP) samples were dried in a vacuum oven at 60 °C overnight. After drying, to encapsulate the carbon shells surrounding the Pt nanoparticles, samples were first kept in a furnace for 1 h in an Ar gas atmosphere at 25 °C. Then, the temperature was raised to 800 °C at a heating rate of 10 °C/min and maintained for 1 h and then allowed to be cooled down. During the temperature ramp-up and maintenance steps, Ar gas and 5 and 10% H_2_/N_2_ mixed gas atmospheres were used, respectively. In particular, the flow rates of 5 and 10% H_2_/N_2_ mixed gases were carefully adjusted to 100 and 200 cm^3^/min to investigate the effect of H_2_ concentration on the carbon shell structure. The prepared catalysts are denoted as Pt@C/C_xHy (x = H_2_ concentration in H_2_/N_2_ mixed gas, y = flow rate of H_2_/N_2_ mixed gas). For instance, Pt@C/C catalysts heat-treated in a 5% H_2_ gas atmosphere with a flow rate of 100 cm^3^/min are named Pt@C/C_5H100.

### 2.2. Physical Characterization

A thermogravimetric analyzer (TGA8000, Woodbridge, ON, USA) was utilized to ascertain the metal loadings of the catalysts. This analysis revealed that all catalysts exhibited comparable metal loadings ranging from 18% to 20% by weight on carbon supports (refer to Figure S1). The average particle sizes and particle size distribution of the prepared catalysts were examined via transmission electron microscopy (TEM) (Tecnai G2 F30 S-Twin, FEI, Eindhoven, The Netherlands). In addition, high-resolution TEM (HR-TEM) (Titan G2 Cube 60–300, FEI, Eindhoven, The Netherlands) facilitated the observation of the carbon shell layer forming the Pt nanoparticles. Furthermore, using an X-ray diffractometer (SmartLab, Rigaku, Tokyo, Japan), the catalysts’ crystal structure was investigated.

### 2.3. Electrochemical Measurements

Electrochemical measurements were conducted within a conventional three-electrode setup employing a rotating disk electrode (RDE) from Metrohm-Autolab, located in Utrecht, The Netherlands, in an argon-saturated 0.1 M HClO_4_ electrolyte solution. The reference and counter electrodes utilized in this work were the Ag/AgCl and Pt wire electrode, respectively. Throughout investigation, applied potentials were referenced to a reversible hydrogen electrode (RHE). The ink of catalysts was formulated by combining 5 mg of the catalyst with 32.3 µL of 5 wt% Nafion solution (Sigma-Aldrich Inc., Burlington, MA, USA) and 500 µL of 99.5% purity 2-propanol (Sigma-Aldrich Inc., Burlington, MA, USA). The metal (38.33 µg/cm^2^) was placed onto a carbon electrode made of glass (GC) of the rotation disk electrode (RDE) by drop-casting the ink of catalysts. Then, the amount of loading was maintained across all samples. Cyclic voltammograms (CVs) were acquired in an electrolyte bubbled with argon, employing a potential scan rate of 20 mV/s over a potential range spanning from 0.05 to 1.05 V. Potential cycling in an oxygen-saturated 0.1 M HClO_4_ electrolyte between 0.05 and 1.05 V at a scan rate of 5 mV/s and a rotation speed of 1600 rpm was used to examine ORR performance. CO stripping curves were conducted in 0.1 M HClO_4_ to evaluate the ECSA. After introducing CO gas into the electrolyte solution for 15 min and holding the working electrode potential at 0.05 V, excess CO molecules were removed by a 20 min argon purge. Subsequently, CVs were recorded in an argon-bubbled electrolyte at a potential scan rate of 20 mV/s within a potential range from 0.05 to 1.05 V. To conclude the experimental procedures, Pt/C (Premetek, Cabot Inc., Boston, MA, USA) was put through 10,000 cycles of accelerated durability testing (ADT) within the potential range of 0.6–1.1 V. These experiments were performed at a scan rate of 100 mV/s in an oxygen-saturated 0.1 M HClO_4_ solution. To determine changes in electrochemical properties, CVs, CO stripping, and ORR were remeasured following ADT.

## 3. Results and Discussion

The preparation of Pt@C/C catalysts involved a simple pyrolysis process followed by an annealing step. Carbon atoms derived from the decomposition of the Pt(acac)_2_ precursor are absorbed into the Pt lattice due to the carbon solubility in Pt. The post-annealing process enables the segregation of absorbed carbon atoms in the metal lattice to encapsulate a carbon shell on the metal surface through graphitization [29,30,31]. In this investigation, the ASP samples were divided into four, and each sample underwent annealing at 800 °C in Ar (0% H_2_) and 5 and 10% H_2_/N_2_ mixed gas conditions with different gas flow rates (100 and 200 cm^3^/min).

The production of significantly larger particles on carbon supports is a result of the well-known severe agglomeration of metal nanoparticles during high-temperature annealing [32]. Nevertheless, despite the high temperature, agglomeration can be effectively avoided if the nanoparticles are coated by carbon shells during the heat treatment process [33]. Figure 1 displays that the carbon shell structure of catalysts can be elaborately designed by simply changing the gas condition duration of the high-temperature annealing process. High-density carbon shells on the surface of the Pt nanoparticles are typically produced by using the non-reactive Ar gas environment. The physicochemical carbon shell structure is observed to be changed by only the thermodynamic driving force during this annealing procedure [34]. Furthermore, similar to the effect of increasing H_2_ concentration in H_2_/N_2_ mixed gas from 5 to 10%, the number of pores (defects) increases, and the thickness of carbon shells gradually decreases when the H_2_/N_2_ gas flow rate rises from 100 to 200 cm^3^/min. A literature survey clearly reveals that H_2_ gas can regulate the growth rate of the carbon layer and generate pores (defects) in graphene by etching the C-C bonds [35,36].

**Figure 2:**
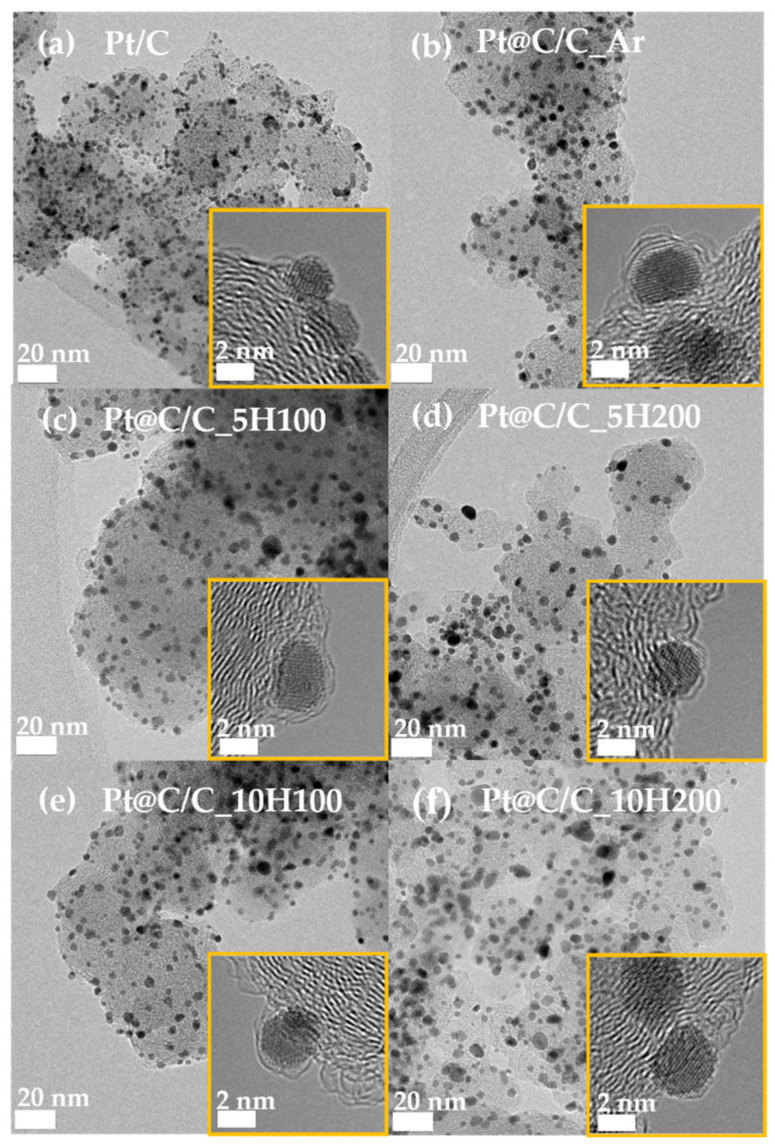
TEM images of (**a**) Pt/C, (**b**) Pt@C/C_Ar, (**c**) Pt@C/C_5H100, (**d**) Pt@C/C_5H200, (**e**) Pt@C/C_10H100, and (**f**) Pt@C/C_10H200. The yellow insets indicate the HR-TEM images of catalysts.

Figure 2 displays TEM images illustrating the particle distribution of samples heat-treated in different gas atmospheres, revealing ultrathin carbon shells encapsulating Pt nanoparticles. It can be confirmed by observing the low-resolution TEM images that the distribution of Pt nanoparticles in all prepared samples is uniform on the carbon supports; about 2–3.5 nm is the average particle size, and it has not changed significantly, as shown in Figures S2 and S3. Furthermore, as illustrated in the insets of Figure 2a–f, the HR-TEM images clearly indicate that ultrathin carbon shell layers are coated onto the Pt nanoparticles during the annealing process, except for a commercial Pt/C catalyst. Meanwhile, even with increasing H_2_ concentration from 5 to 10%, the average particle sizes of the catalysts hardly change. Contrary to this, Pt@C/C_5H200 and Pt@C/C_10H200 catalysts annealed under higher gas flow conditions of 200 cm^3^/min have slightly increased and agglomerated particles owing to the encapsulation of thinner carbon layers by the accelerated etching process. Nevertheless, identifying the detailed information about the thickness and pore structure of the carbon shells is difficult owing to the limitations of TEM analysis.

The X-ray diffraction (XRD) patterns of heat-treated Pt@C/C catalysts were obtained to further understand the crystal structure (Figure S4). All the Pt/C and Pt@C/C catalysts exhibit the same 2θ degree values of 39.7, 46.2, and 67.4°, corresponding to the (111), (200), and (220) facets of the typical Pt fcc structure, respectively (JCPDS card No.: 00-004-0802) [37]. However, as the H_2_/N_2_ mixed gas flow rate and concentration increase, the peaks of the samples slightly become sharper, showing the slight change in crystallite size. As a result, both the change in the mean particle size calculated from the TEM images and the average crystallite size assessed from the full-width half maximum (FWHM) of the (220) XRD peak demonstrate a similar trend (Table S1).

**Figure 3:**
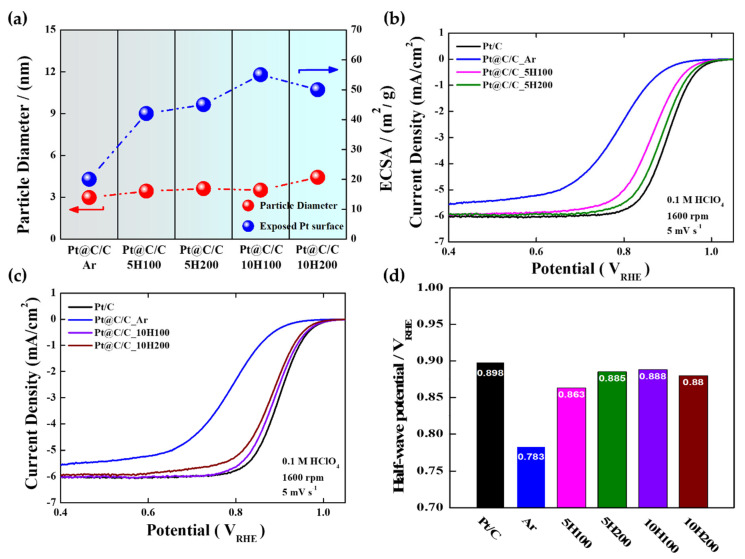
(**a**) Correlation between particle diameters and ECSAs of Pt@C/C_Ar, Pt@C/C_5H100, Pt@C/C_5H200, Pt@C/C_10H100, and Pt@C/C_10H200; ORR polarization curves of Pt@C/C catalysts annealed in (**b**) 5% H_2_ and (**c**) 10% H_2_ gas atmospheres; (**d**) half-wave potentials of Pt/C, Pt@C/C_Ar, Pt@C/C_5H100, Pt@C/C_5H200, Pt@C/C_10H100, and Pt@C/C_10H200 catalysts.

The CO stripping and CV curves were utilized to indirectly estimate the surface coverage of the carbon shells. (Figure S5). The CO stripping curves were employed to calculate the ECSAs of the catalysts. In fact, the ECSA of the samples can be proportional to the Pt surface area exposed though the carbon shell layer. Accordingly, it can be concluded that the ECSA is closely related to the change in carbon shell structure (for instance, the surface coverage), indicating that the more porous the carbon shells, the higher the ECSA. As expected, the Pt@C/C_Ar catalyst has a much smaller ECSA value of 20 m^2^/g as a result of the dense and thick carbon shell forming. As shown in Figure 3a, it is revealed that with increasing the H_2_ content or flow rate in the annealing process, the ECSA increases, while the average particle size hardly changes [38]. However, the Pt@C/C_10H200 catalyst exhibits a slightly lower ECSA (50 m^2^/g) in contrast to the Pt@C/C_10H100 catalyst (55 m^2^/g) since the Pt nanoparticles can be agglomerated due to the much-reduced thickness of the carbon shell at higher H_2_ gas concentrations. Considering the correlation between the extent of carbon shell coverage and the composition of the annealing gas, it can be inferred that carbon etching by H_2_ is efficient in fine-tuning the structure of the carbon shell while minimizing the risk of significant particle agglomeration when selecting the appropriate H_2_ content and flow rate.

As illustrated in Figure 3b,c, except for Pt@C/C_10H200, the ORR polarization curves of Pt@C/C catalysts annealed in 5 and 10% H_2_ gas atmospheres strongly support that the overall electrocatalytic activity increases with the ECSA. In other words, increasing the H_2_ content to expose more metal surface through the carbon etching effect leads to gradually improved catalytic activity. Firstly, the Pt@C/C_Ar catalyst impedes O_2_ penetration through the carbon shell with a higher density [39], resulting in the lowest ORR activity among the prepared samples (Figure 3d). The Pt@C/C_10H200 catalyst exhibits a relatively reduced activity compared to the Pt@C/C_10H100 catalyst, which is primarily due to the presence of agglomerated particles leading to a decrease in the ECSA. Conversely, the ORR performance of the Pt@C/C_5H100 catalyst was found to be inadequate, displaying notably lower activity in comparison with the Pt/C catalyst owing to the presence of denser carbon layers potentially impeding activity compared to Pt@C/C_5H200. Therefore, the rationale behind selecting the optimized Pt@C/C_10H100 catalyst rather than the Pt@C/C_5H200 catalyst even with comparable activity to further investigate the impact of the carbon shell’s pore structure on durability stems from its superior ECSA, mass activity, and half-wave potential (Table 1 and Table S2). Meanwhile, the Tafel plots for the ORR were generated by correlating the kinetic current densities with the electrode potential (Table 1 and Figure S6). As a result, the Tafel slopes of Pt/C, Pt@C/C_Ar, Pt@C/C_5H100, Pt@C/C_5H200, Pt@C/C_10H100, and Pt@C/C_10H200 were determined to be 58, 81, 62, 60, 59, and 63 mV/dec, respectively (Figure S6). It is anticipated that a Tafel slope nearing 60 mV represents the anticipated magnitude for a single-electron charge transfer functioning as the rate-determining step (RDS). Pt@C/C_Ar exhibits a higher Tafel slope in comparison to Pt/C as well as other samples which can be attributed to a lower active area because of a thick, dense carbon shell, resulting in poor ORR kinetics. These changes in the Tafel slope are caused by the ORR passing through the PtOx species, primarily PtO and Pt-OH, and then reducing such Pt oxides to a free-oxide Pt surface [40,41,42,43]. In addition, the electron transfer numbers (n) for the ORR were calculated from the Koutecky–Levich plots (Figures S7 and S8), demonstrating that the ORR for all Pt catalysts predominantly follows a 4e-pathway.

**Figure 4:**
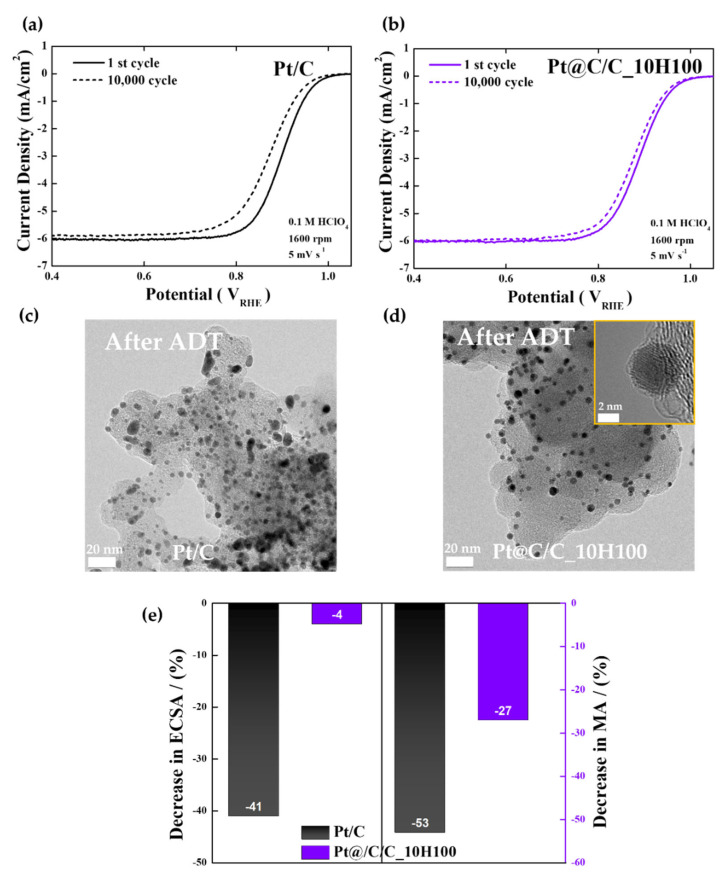
ADT results. (**a**,**b**) Change in ORR polarization curves of Pt/C and Pt@C/C_10H100 catalysts before and after ADTs. (**c**,**d**) TEM images of Pt/C and Pt@C/C_10H100 catalysts after ADTs. (**e**) Change in ECSAs and mass activities of Pt/C and Pt@C/C_10H100 catalysts after ADTs. Yellow inset indicates HR-TEM image of Pt@C/C_10H100 catalysts.

Lastly, O_2_-saturated 0.1 M HClO_4_ solutions were utilized for ADTs in the potential range of 0.6−1.1 V involving 10,000 potential cycles to evaluate the durability of the commercial Pt/C and Pt@C/C_10H100 catalysts (Figure 4). Figure 4a illustrates how the Pt/C catalyst shows high ORR activity at first, but following 10,000 cycles, it considerably decreases. Based on the TEM image after the ADT, as shown in Figure 4c, it is thought that the performance degradation is caused by the severe agglomeration of Pt nanoparticles (Figure S9). Contrary to this, as shown in Figure 4b, the Pt@C/C_10H100 catalyst shows superior stability to Pt/C even after 10,000 cycles, and only a 12 mV drop is observed in the catalyst’s half-wave potential (E_1/2_) compared to the initial one (Table S3). The TEM images taken after the ADT (the inset of Figure 4d) definitely show that the distribution and size of Pt nanoparticles hardly changed because of the presence of robust carbon shell layers (Figure S9).

As displayed in Figure 4e and Figure S10, the ECSA of the Pt/C catalyst indicates a sharp drop in the number of active sites (−41%) following the ADT compared to the initial one mainly due to the structural degradation of Pt nanoparticles. Conversely, the ECSA of the Pt@C/C_10H100 catalyst hardly changes after the ADT (−4%). Furthermore, the decrease in mass activity at 0.9 V is much more severe for Pt/C than Pt@C/C_10H100, implying that the well-controlled carbon shells can contribute positively to the long-term stability of fuel cell catalysts.

## 4. Conclusions

The significant role of heat treatment conditions has been investigated in this study, especially H_2_ concentration, which can be regulated by changing the H_2_ content and gas flow rate of the H_2_/N_2_ mixed gas to fabricate the finely tuned carbon shell structure on the metal nanoparticles. Using only a small quantity of the carbon source in the Pt(acac)_2_ precursor, the Pt@C/C catalysts with various carbon shell structures were effectively synthesized through thermal decomposition and subsequent annealing without the need for further polymer coating. In addition, the highly active and durable Pt@C/C catalyst with ultrathin carbon shells was developed by elaborately optimizing the gas annealing conditions such as gas flow rates and the H_2_ content. Consequently, this study suggests that balancing the flow rate and H_2_ content of the H_2_/N_2_ mixed gas is a key parameter in designing the best structure of the carbon shells to develop promising fuel cell catalysts.

## Figures and Tables

**Table 1:** ECSA, half-wave potential, mass activity, and Tafel slope results for Pt/C, Pt@C/C_Ar, Pt@C/C_5H100, Pt@C/C_5H200, Pt@C/C_10H100, and Pt@C/C_10H200 catalysts.

Catalysts	ECSA (m^2^/g)	Half-Wave Potential (V_RHE_)	Mass-Activity (A/mg) at 0.9 V	Tafel Slopes (mV/dec)
Pt/C	99	0.898	0.148	58
Pt@C/C_Ar	20	0.783	0.009	81
Pt@C/C_5H100	42	0.863	0.054	62
Pt@C/C_5H200	45	0.885	0.098	60
Pt@C/C_10H100	55	0.888	0.110	59
Pt@C/C_10H200	50	0.88	0.088	63

## Data Availability

Data are contained within the article and Supplementary Materials.

## Supplementary Materials

The following supporting information can be downloaded at https://www.mdpi.com/article/10.3390/nano14110924/s1, Figure S1: TGA curves of (a) Pt/C_ASP, (b) Pt@C/C_Ar, (c) Pt@C/C_5H100, (d) Pt@C/C_5H200, (e) Pt@C/C_10H100, and (f) Pt@C/C_10H200 catalysts; Figure S2: TEM, HR-TEM images, and particle size distribution of (a–c) Pt/C, (d–f) Pt/C_ASP, (g–i) Pt@C/C_Ar catalysts; Figure S3: TEM, HR-TEM images, and particle size distribution of (a–c) Pt@C/C_5H100, (d–f) Pt@C/C_5H200, (g–f) Pt@C/C_10H100, and (j–l) Pt@C/C_10H200 catalysts; Figure S4: XRD patterns of Pt/C, Pt/C_ASP, Pt@C/C_Ar, Pt@C/C_5H100, Pt@C/C_5H200, Pt@C/C_10H100, and Pt@C/C_10H200 catalysts; Figure S5: CVs and CO stripping curves of (a,b) Pt@C/C_Ar, Pt@C/C_5H100, and Pt@C/C_5H200, (c,d) Pt@C/C_Ar, Pt@C/C_10H100, and Pt@C/C_10H200. For comparison, the results of Pt/C are represented in each figure; Figure S6: The mass transport corrected Tafel plots of (a) Pt/C, Pt@C/C_Ar, Pt@C/C_5H100, and Pt@C/C_5H200 (b) Pt/C, Pt@C/C_Ar, Pt@C/C_10H100, and Pt@C/C_10H200 catalysts; Figure S7: Catalysts in an O_2_-saturated 0.1 M HClO_4_ solution at a scanning rate of 5 mV/s at various rotation rates ranging from 400 to 2500 rpm, (a) Pt/C, (b) Pt/C_ASP, (c) Pt@C/C_Ar, (d) Pt@C/C_5H100, (e) Pt@C/C_5H200, (f) Pt@C/C_10H100, and (g) Pt@C/C_10H200; Figure S8: Koutecky–Levich plots of ORR on (a) Pt/C, (b) Pt/C_ASP, (c) Pt@C/C_Ar, (d) Pt@C/C_5H100, (e) Pt@C/C_5H200, (f) Pt@C/C_10H100, and (g) Pt@C/C_10H200 catalysts at different potentials; Figure S9: TEM images and particle size distribution of (a,b) Pt/C and (c,d) Pt@C/C_10H100 catalysts after ADTs. (e) HR-TEM image of Pt@C/C_10H100 catalyst after ADTs; Figure S10: CVs and CO stripping curves of (a,b) Pt/C, (c,d) Pt@C/C_10H100 before and after ADTs; Table S1: XRD crystallite size and TEM mean particle size results for Pt/C, Pt/C_ASP, Pt@C/C_Ar, Pt@C/C_5H100, Pt@C/C_5H200, Pt@C/C_10H100, and Pt@C/C_10H200 catalysts; Table S2: A comparison of the electrochemical properties of Pt/C catalysts based on a literature survey; Table S3: Change in ECSA, half-wave potential, and mass activity of Pt/C and Pt@C/C_10H100 catalysts before and after ADTs. Refs. [44,45,46,47,48,49,50,51,52,53] are cited in Supplementary Materials.
