# Supplementary material for: Effect of Heat Treatment on Structure of Carbon Shell-Encapsulated Pt Nanoparticles for Fuel Cells
Source: Nanomaterials (Basel). 2024 May 24;14(11):924. doi: 10.3390/nano14110924 (PMC11173419; doi:10.3390/nano14110924)
Supplement: Supplementary file 1 [file nanomaterials-14-00924-s001.zip › nanomaterials-2966682-supplementary.pdf]

Supplementary Information

Effect of Heat Treatment on Structure of Carbon Shell-Encapsulated Pt Nanoparticles for Fuel Cells

Khikmatulla Davletbaev[1,2], Sourabh S. Chougule[1], Jiho Min[1], Keonwoo Ko[1], Yunjin Kim[1], Hyeonwoo Choi[1], Yoonseong Choi[1], Abhishek A. Chavan[1], Beomjun Pak[1], Ikromjon U. Rakhmonov[2,*] and Namgee Jung[1,*]

[1]Graduate School of Energy Science and Technology (GEST), Chungnam National University, 99 Daehak-ro, Yuseong-gu, Daejeon 34134, Republic of Korea; haki030899@o.cnu.ac.kr (K.D.); schougule@o.cnu.ac.kr (S.S.C.); mjh9780@o.cnu.ac.kr (J.M.); kkw00000@o.cnu.ac.kr (K.K.); yunjinkim1994@o.cnu.ac.kr (Y.K.); snow7780@o.cnu.ac.kr (H.C.); gubongman@o.cnu.ac.kr (Y.C.); abhishek@o.cnu.ac.kr (A.A.Ch.); ssansai@o.cnu.ac.kr (B.J.P.)

[2]Department of Power Supply, Tashkent State Technical University, Tashkent 100095, Uzbekistan

*Correspondence: njung@cnu.ac.kr (N.J.); ilider1987@yandex.ru (I.U.R.)

Experimental

All electrochemical experiments data were obtained by using Ag/AgCl reference electrode. To convert from Ag/AgCl electrode voltage to the reversible hydrogen electrode (RHE) voltage, the calculation method of voltage conversion was used. In this study, electrolyte solution is 0.1 M $HClO_4$, pH value is 1.2.

$$E\ (vs.\ RHE) = E\ (vs.\ Ag/AgCl) + 0.210 + 0.059\ pH$$

Electrochemical active surface areas (ECSAs) of catalysts were calculated from CO stripping curves using the following equation.

$$ECSA(m^2/g) = \frac{\textbf{Total Charge }(Q)\textbf{ for CO oxidation}}{Q_{CO}(420\mu C\ cm^{-2})\ x\ Pt\ loading(gpt)}$$

Polarization plots were recorded in O_2-saturated electrolyte at scan rate of 5 mV/s. The disk rotation rate was varied from 400 to 2500 rpm. Koutecky-Levich plots (J^{-1} vs. $\omega^{-1/2}$) were analyzed at various electrode potentials. The slopes of their best linear fit lines were used to calculate the number of electrons transferred (n) on the basis of the Koutecky-Levich equation.

$$\frac{1}{J_L} = \frac{1}{J_L} + \frac{1}{J_K} = \frac{1}{B\omega^{1/2}} + \frac{1}{J_K}$$

$$B = 0.62nFC_0(D_0)^{2/3}v^{-1/6} \qquad J_K = nFkC_0$$

Where J is the measured current density, J_K and J_L are the kinetic and diffusion-limiting current densities, ω is the angular velocity, n is transferred electron number, F is the Faraday constant, C_0 is the bulk concentration of O_2, v is the kinematic viscosity of the electrolyte, and k is the electron-transfer rate constant.

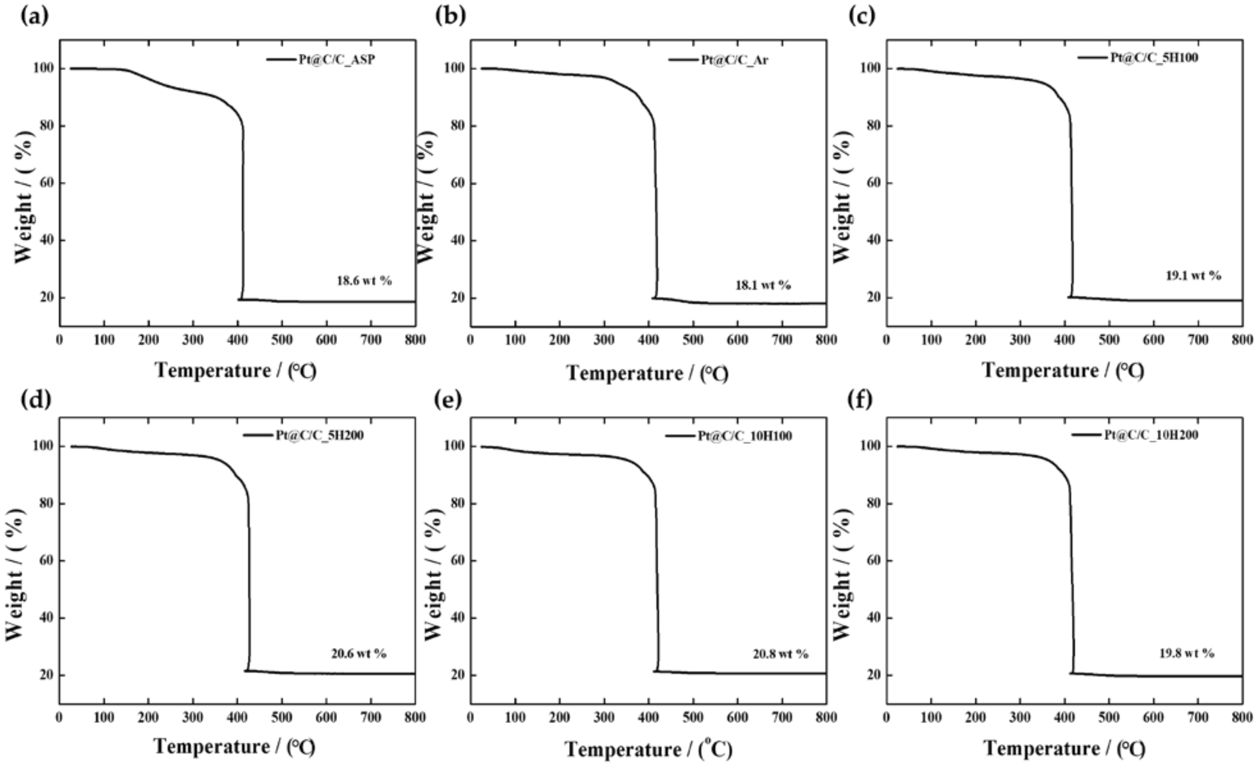

Figure S1. TGA curves of (**a**) Pt/C_ASP (**b**) Pt@C/C_Ar, (**c**) Pt@C/C_5H100, (**d**) Pt@C/C_5H200, (**e**) Pt@C/C_10H100, (**f**) Pt@C/C_10H200 catalysts.

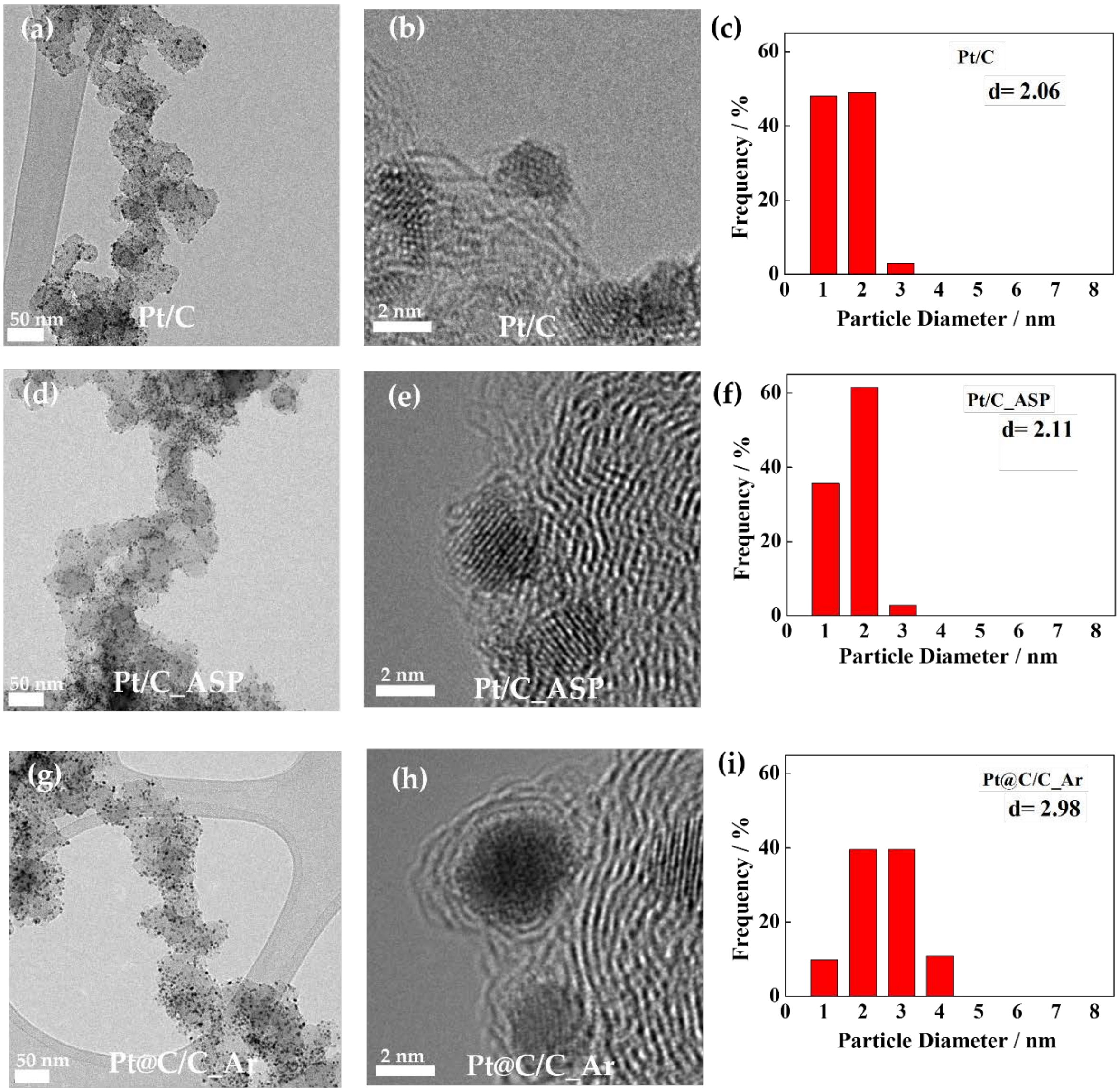

Figure S2. TEM, HR-TEM images, and particle size distribution of (**a-c**) Pt/C, (**d-f**) Pt/C_ASP, (**g-i**) Pt@C/C_Ar catalysts.

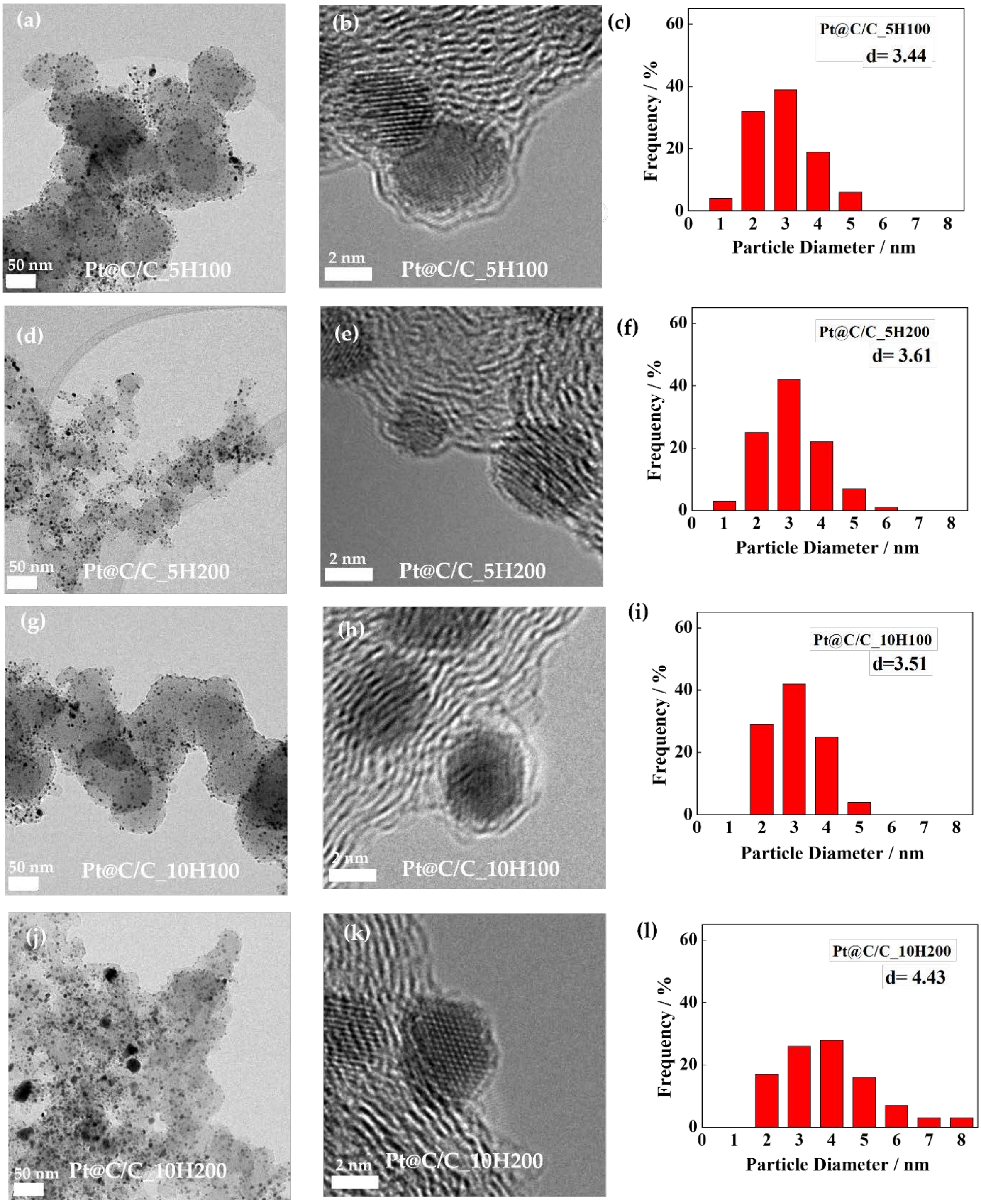

Figure S3. TEM, HR-TEM images, and particle size distribution of (**a-c**) Pt@C/C_5H100, (**d-f**) Pt@C/C_5H200, (**g-f**) Pt@C/C_10H100, (**j-l**) Pt@C/C_10H200 catalysts.

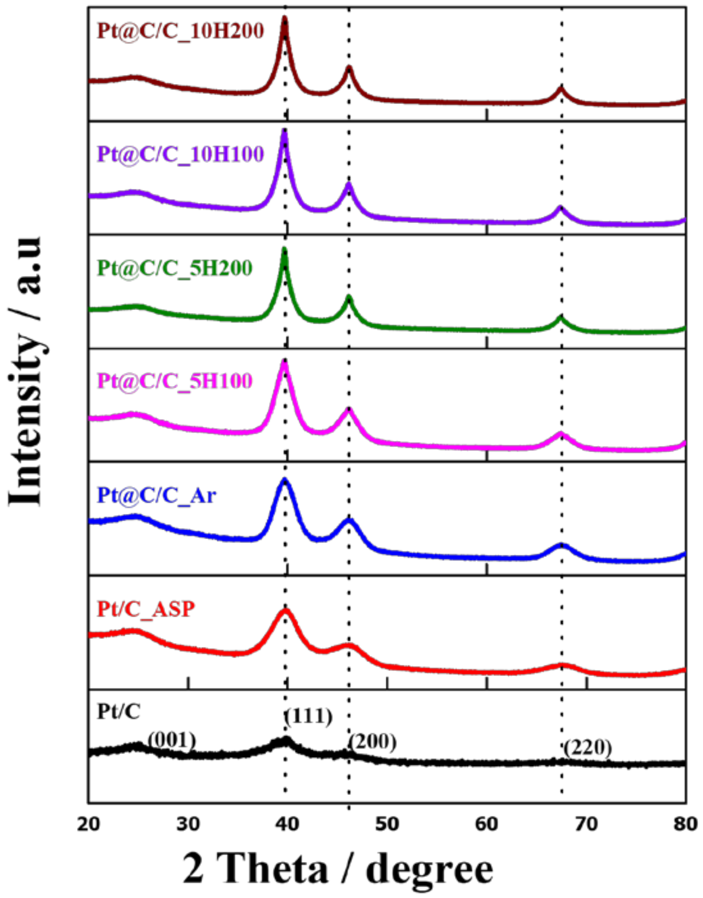

Figure S4. XRD patterns of Pt/C, Pt/C_ASP, Pt@C/C_Ar, Pt@C/C_5H100, Pt@C/C_5H200, Pt@C/C_10H100, and Pt@C/C_10H200 catalysts.

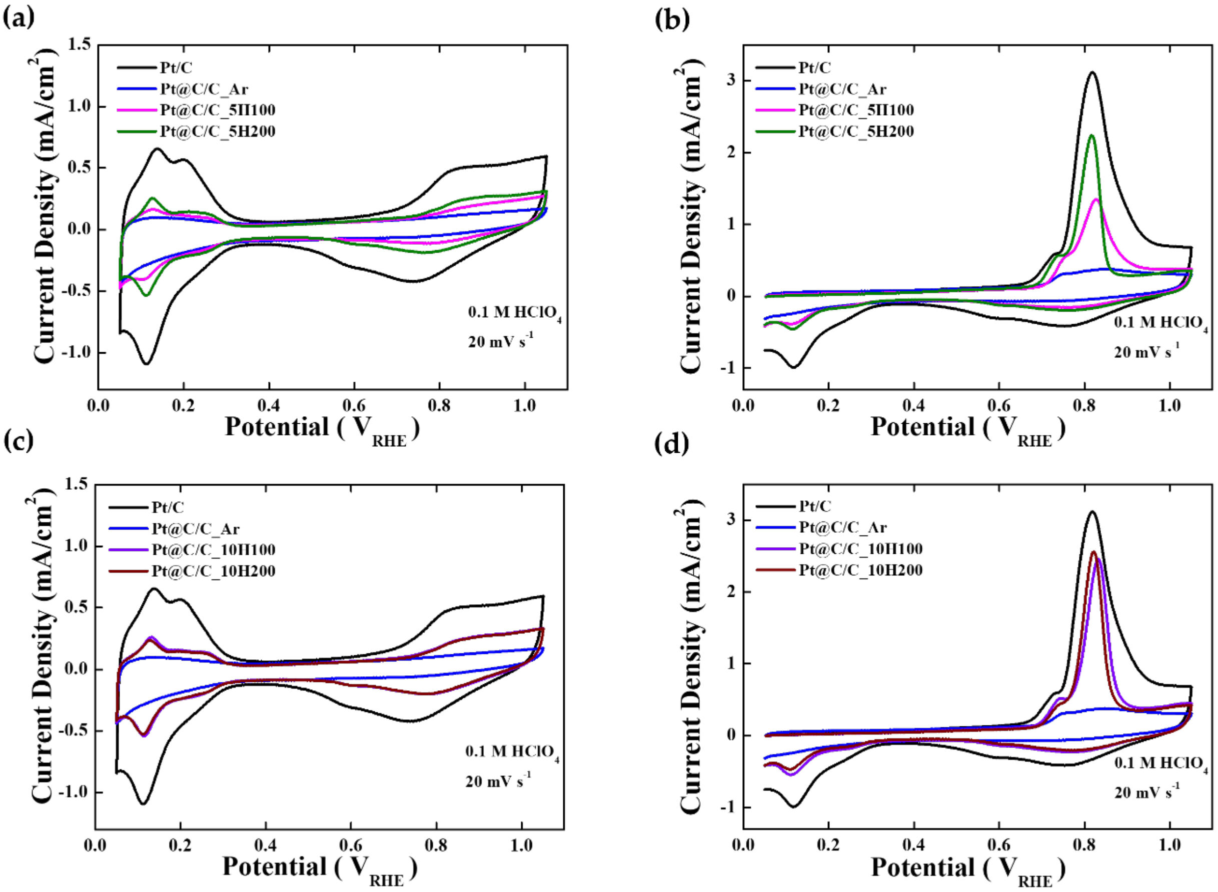

Figure S5. CVs and CO stripping curves of (**a**-**b**) Pt@C/C_Ar, Pt@C/C_5H100, and Pt@C/C_5H200, (**c**-**d**) Pt@C/C_Ar, Pt@C/C_10H100, and Pt@C/C_10H200. For comparison, the results of Pt/C are represented in each figure.

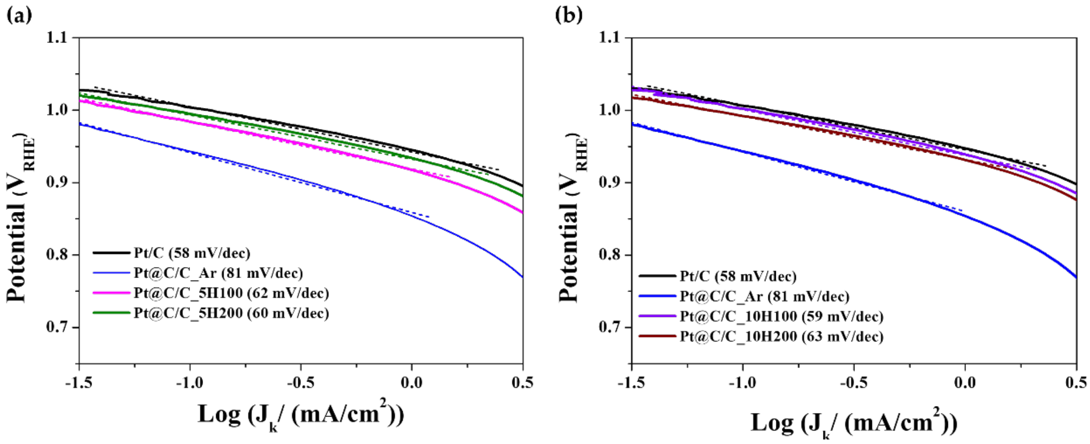

Figure S6. The mass transport corrected Tafel plots of (**a**) Pt/C, Pt@C/C_Ar, Pt@C/C_5H100 and Pt@C/C_5H200 (**b**) Pt/C, Pt@C/C_Ar, Pt@C/C_10H100 and Pt@C/C_10H200 catalysts.

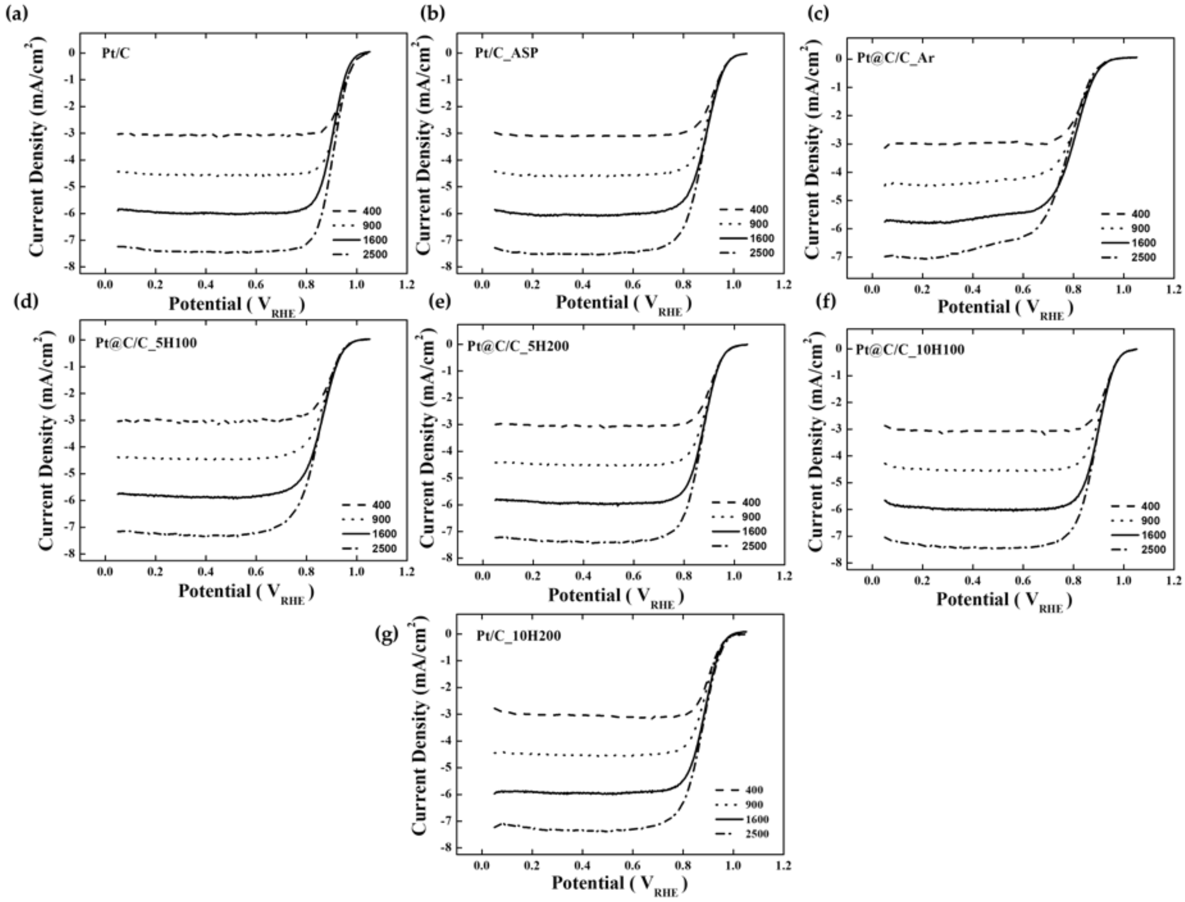

Figure S7. Catalysts in O_2 saturated 0.1 M $HClO_4$ solution at a scanning rate of 5 mV/s at various rotation rates ranging from 400 to 2500 rpm, (**a**) Pt/C, (**b**) Pt/C_ASP, (**c**) Pt@C/C_Ar, (**d**) Pt@C/C_5H100, (**e**) Pt@C/C_5H200, (**f**) Pt@C/C_10H100 and (**g**) Pt@C/C_10H200.

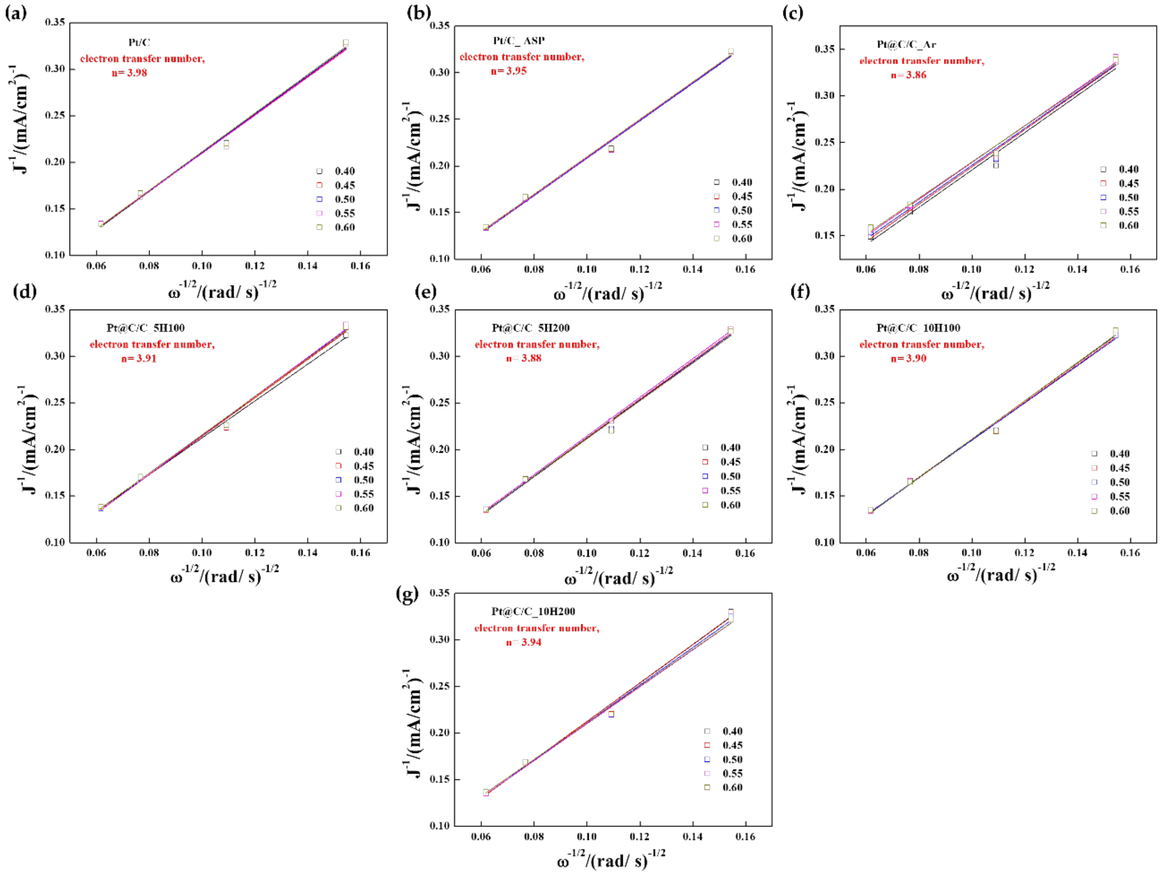

Figure S8. Koutecky-Levich plots of ORR on (**a**) Pt/C, (**b**) Pt/C_ASP, (**c**) Pt@C/C_Ar, (**d**) Pt@C/C_5H100, (**e**) Pt@C/C_5H200, (**f**) Pt@C/C_10H100, (**g**) Pt@C/C_10H200 catalysts at different potentials.

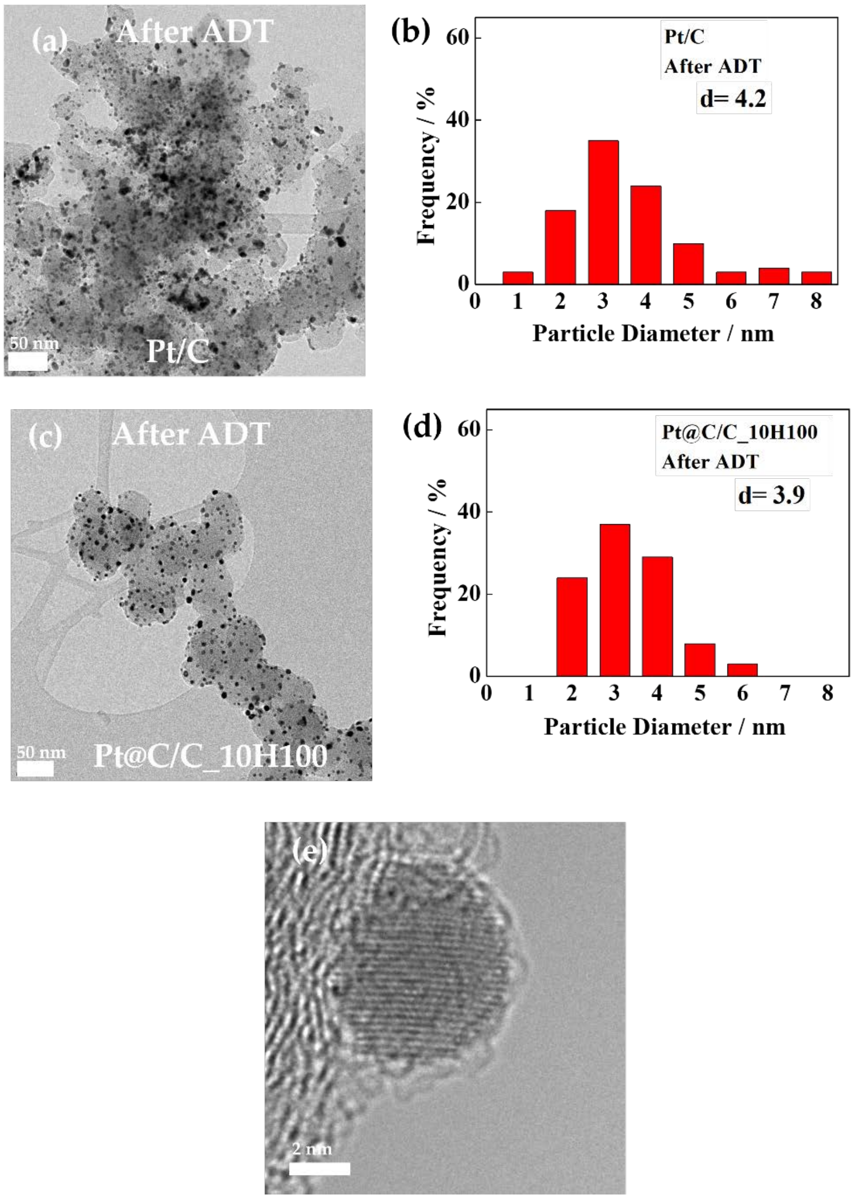

Figure S9. TEM images and particle size distribution of (**a-b**) Pt/C and (**c-d**) Pt@C/C_10H100 catalysts after ADTs. (**e**) HR-TEM image of Pt@C/C_10H100 catalyst after ADTs.

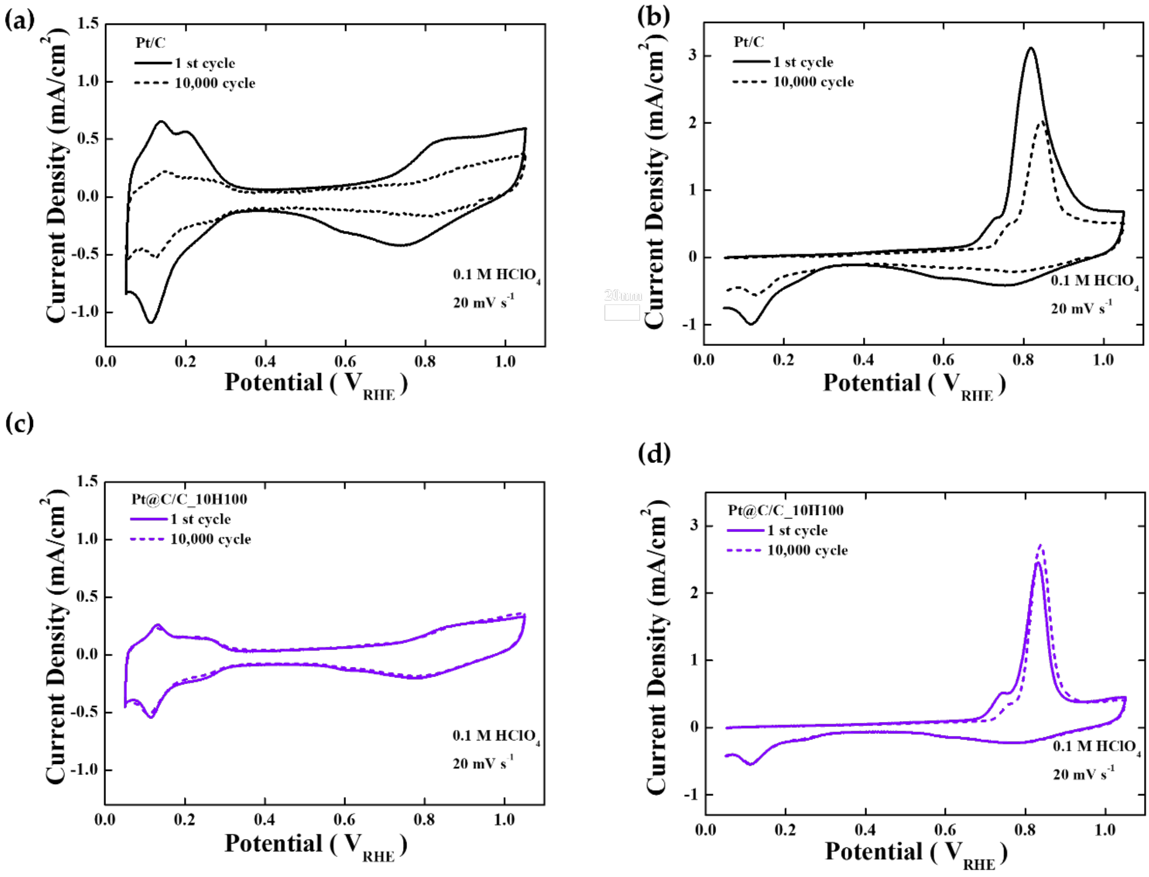

Figure S10. CVs and CO stripping curves of (**a**-**b**) Pt/C and (**c**-**d**) Pt@C/C_10H100 catalysts before and after ADTs.

Table S1. XRD crystallite size and TEM mean particle size results for Pt/C, Pt/C_ASP, Pt@C/C_Ar, Pt@C/C_5H100, Pt@C/C_5H200, Pt@C/C_10H100, and Pt@C/C_10H200 catalysts.

Catalysts	XRD (Crystalline size)	TEM (Particle size)
**Pt/C**	1.63 nm	2.06 nm
**Pt/C_ASP**	2.05 nm	2.11 nm
**Pt@C/C_Ar**	2.93 nm	2.98 nm
**Pt@C/C_5H100**	3.66 nm	3.44 nm
**Pt@C/C_5H200**	4.81 nm	3.61 nm
**Pt@C/C_10H100**	4.74 nm	3.51 nm
**Pt@C/C_10H200**	5.24 nm	4.43 nm

Table S2. Comparison of the electrochemical properties of Pt/C catalysts based on a literature survey.

Catalysts (20 wt%)	Electrolyte	Loading (μg/cm^2)	ECSA (m^2/g)	Scan rate (ORR) (mV/s)	Mass-activity at 0.9V (A/mg)	Reference
**Pt/C**	0.1 M $HClO_4$	38.33	99	5	0.148	In this work
**Pt/C**	0.1 M $HClO_4$	40.6	65	10	0.13	[1]
**Pt/C**	0.1 M $HClO_4$	12.75	65	10	0.143	[2]
**Pt/C**	0.1 M $HClO_4$	30.61	54.3	5	0.14	[3]
**Pt/C**	0.1 M $HClO_4$	17.9	60.9	10	0.180	[4]
**Pt/C**	0.1 M $HClO_4$	6.4	69.3	10	0.130	[5]
**Pt/C**	0.1 M $HClO_4$	-	-	5	0.160	[6]
**Pt/C**	0.1 M $HClO_4$	16	-	10	0.140	[7]
**Pt/C**	0.1 M $HClO_4$	20	51	5	0.130	[8]
**Pt/C**	0.1 M $HClO_4$	36.7	100	20	0.156	[9]
**Pt/C**	0.1 M $HClO_4$	-	-	-	0.130	[10]

Table S3. Change in ECSA, half-wave potential, and mass activity of Pt/C and Pt@C/C_10H100 catalysts before and after ADTs.

Catalysts	Before ADT			After ADT		
	ECSA (m^2/g)	Half-wave potential (V_{RHE})	Mass-activity at 0.9V (A/mg)	ECSA (m^2/g)	Half-wave potential (V_{RHE})	Mass-activity at 0.9V (A/mg)
**Pt/C**	99	0.898	0.148	55	0.873	0.069
**Pt@C/C_10H100**	55	0.888	0.110	53	0.876	0.08

References

1. Xia, Y. F.; Guo, P.; Li, J. Z.; Zhao, L.; Sui, X.L.; Wang, Y.; Wang, Z. B. How to appropriately assess the oxygen reduction reaction activity of platinum group metal catalysts with rotating disk electrode. *IScience* **2021**, *24*.
2. Liu, Y.; Wu, M.; Sheng, S.; Zhi, C.; Wang, Y.; Li, X.; The fabrication of Pt–Pb alloy networks with high-density micropores by dealloying for enhanced oxygen reduction activity. *International Journal of Hydrogen Energy* 2023, 48, pp.13470-13478.
3. Chen, Z.; Shengli C. Facile synthesis of platinum-copper aerogels for the oxygen reduction reaction. Energy Mater **2022**, *2*, 10-20517.
4. Huang, H.; Li, K.; Chen, Z.; Luo, L.; Gu, Y.; Zhang, D.; Ma, C.; Si, R.; Yang, J.; Peng, Z.; Zeng, J. Achieving remarkable activity and durability toward oxygen reduction reaction based on ultrathin Rh-doped Pt nanowires. *Journal of the American Chemical Society* **2017**, *139*, pp.8152-8159.
5. Jiang, K.; Zhao, D.; Guo, S.; Zhang, X.; Zhu, X.; Guo, J.; Lu, G.; Huang, X. Efficient oxygen reduction catalysis by subnanometer Pt alloy nanowires. *Science Advances* **2017**, *3*, p.e1601705.
6. He, D. S.; He, D.; Wang, J.; Lin, Y.; Yin, P.; Hong, X.; Wu, Y.; Li, Y. Ultrathin icosahedral Pt-enriched nanocage with excellent oxygen reduction reaction activity. *Journal of the American Chemical Society* **2016**, *138*, pp.1494-1497.
7. Chung, D. Y.; Jun, S. W.; Yoon, G., Kwon; S.G.; Shin, D.Y.; Seo, P.; Yoo, J. M.; Shin, H.; Chung, Y. H.; Kim, H.; Mun, B. S. Highly durable and active PtFe nanocatalyst for electrochemical oxygen reduction reaction. *Journal of the American Chemical Society* **2015**, *137*, pp.15478-15485.
8. Zhu, J.; Xiao, M.; Zhao, X.; Liu, C.; Ge, J.; Xing, W. Strongly coupled Pt nanotubes/N-doped graphene as highly active and durable electrocatalysts for oxygen reduction reaction. *Nano Energy* **2015**, *13*, pp.318-326.0.
9. Alekseenko, A. A.; Guterman, V. E.; Belenov, S. V.; Menshikov, V. S.; Tabachkova, N. Y.; Safronenko, O. I.; Moguchikh, E. A. Pt/C electrocatalysts based on the nanoparticles with the gradient structure. International *Journal of Hydrogen Energy* **2018**, *43*, pp.3676-3687.
10. Zhang, H.; Yi, B.; Jiang, S.; Zeng, Y.; Shao, Z. Three-Dimensional Assembly of PtNi Alloy Nanosticks with Enhanced Electrocatalytic Activity and Ultrahigh Stability for the Oxygen Reduction Reaction. *ChemElectroChem* **2017**, *4*, pp.1436-1442.
